# Laceration of the Cervix a Cause of Sterility, Miscarriage, Etc.

**Published:** 1879-03

**Authors:** 


Laceration of the Cervix a Cause of Sterilíay, Miscarriage, Etc.—This lesion is so common a cause of sterility that I always suspect its existence whenever a guileless woman stops bearing after her first labor. The sterility is due partly, of course, to the disorders, the flexions, and dislocations of the womb which, as I have shown, follow such an injury. But it is due also to the acridity of the discharges, which kills the spermatozoa, or to the viscious plug of mucus which often closes the remnant of the cervical canal. Again, the deep notches in the cervix hinder that suction action of the womb during the sexual orgasm, just as the split nozzle of a syringe cannot suck up a thin stratum of fluid. Further, the cervical canal denuded of its epithelium presents such a barrier to the migration of the spermatozoa as a desert does to the advance of an army. But these are not the only evils following such an injury. The weakened retentive power of the cervix often leads to repeated miscarriages. This I have known to happen over and over again. Often have I been obliged to puncture or to cross-hatch a brood of retention cysts which aided in the eversion of the mucous lining. Once I removed a sessile polypus as large as a pigeon's egg, which grew out of a cluster of exposed Nabothian glands. Further, I feel very sure that many an epithelial cancer of the cervix starts from such a constantly chafed and fretted surface; for, in my experience, a cancer of even a movable womb with a ragged notch on one side of the cervix apparently eaten down to the vaginal junction, is no uncommon event.—Goodell, *Penn. State Society's Transactions.*

